# Prolonged Survival of NUT Midline Carcinoma and Current Approaches to Treatment

**DOI:** 10.1093/oncolo/oyad177

**Published:** 2023-06-13

**Authors:** Henry G Kaplan, Somasundaram Subramaniam, Eric Vallières, Todd Barnett

**Affiliations:** Medical Oncology, Swedish Cancer Institute, Seattle, WA, USA; Medical Oncology, Swedish Cancer Institute, Seattle, WA, USA; Medical Oncology, Swedish Cancer Institute, Seattle, WA, USA; Medical Oncology, Swedish Cancer Institute, Seattle, WA, USA

**Keywords:** Nut midline carcinoma, BET, BRD

## Abstract

NUT midline carcinoma is a rare malignancy most commonly seen in adolescents and young adults. The disease presents most often in the lung or head and neck area but can be seen occasionally elsewhere. The diagnosis can be difficult and requires a high degree of suspicion with demonstration of the classic fusion rearrangement mutation of the *NUTM1* gene with one of a variety of partners by immunohistochemistry, fluorescent in situ hybridization, or genomic analysis. Survival is usually only a number of months with few long-term survivors. Here we report one of the longest-known survivors of this disease treated with surgery and radiation without additional therapy. Systemic treatment approaches including the use of chemotherapy and BET and histone deacetylase inhibitors have yielded modest results. Further studies of these, as well as p300 and CDK9 inhibitors and combinations of BET inhibitors with chemotherapy or CDK 4/6 inhibitors, are being evaluated. Recent reports suggest there may be a role for immune checkpoint inhibitors, even in the absence of high tumor mutation burden or PD-L1 positivity. RNA sequencing of this patient’s tumor demonstrated overexpression of multiple potentially targetable genes. Given the altered transcription that results from the causative mutation multi-omic evaluation of these tumors may uncover druggable targets for treatment.

## Teaser

NUT midline carcinoma is a rare malignancy that can be difficult to diagnose. Survival is usually only a number of months with few long-term survivors. This article reports one of the longest-known survivors of this disease treated with surgery and radiation without additional therapy.

**Key Points**

- NMC is a rare malignancy of adolescents and young adults that requires specialized pathological and genomic studies for diagnosis.
- Treatment involves surgical resection and radiation. Multiple systemic therapies are being used but there is no clear standard of care for this tumor.
- Additional multi-omic research is needed to develop targeted systemic therapies.

## Patient Story

**Figure 1.**
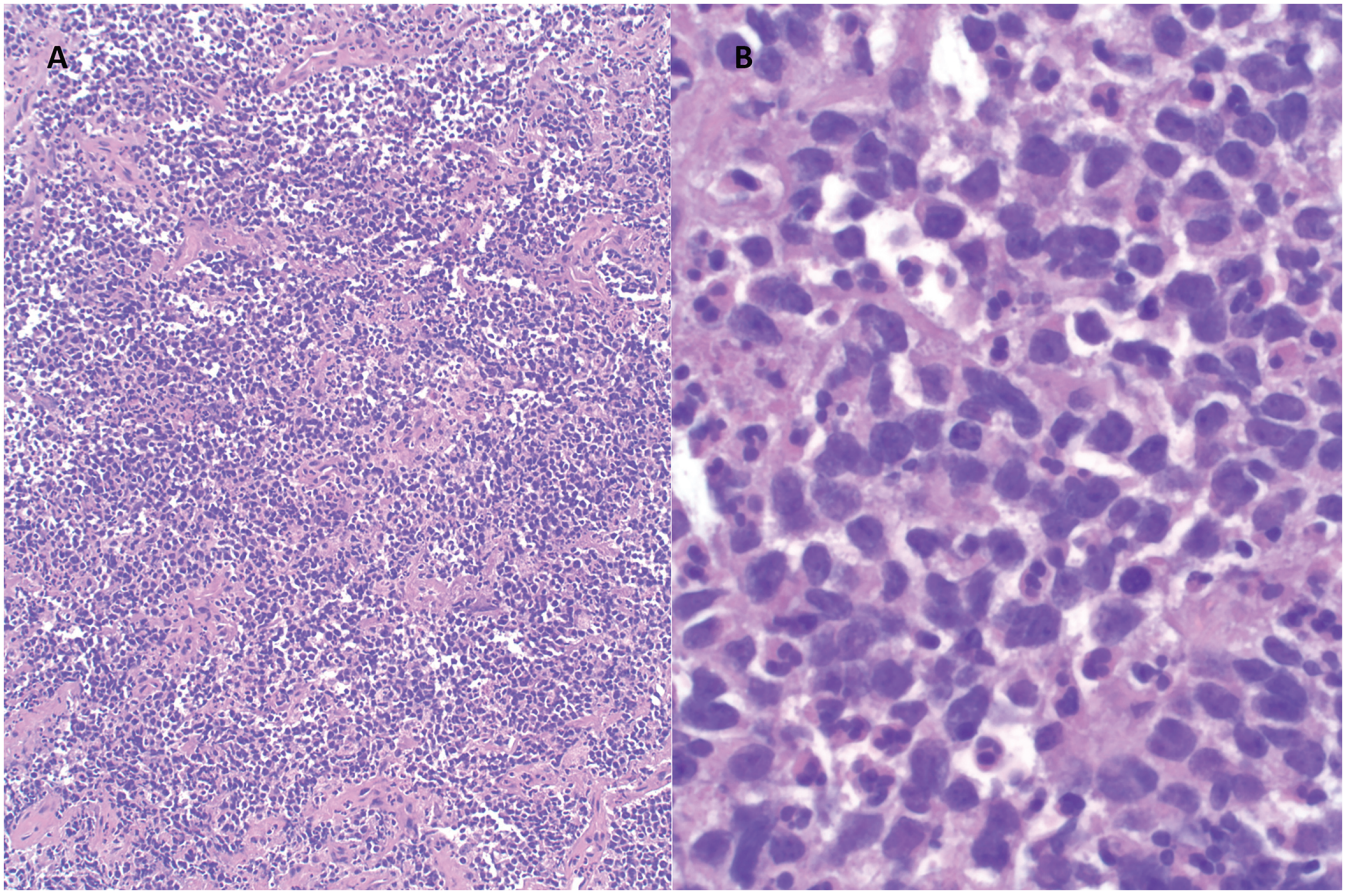
Pathology of NMC. H&E stain, (**A**) 10× and (**B**) 60×.

**Figure 2.**
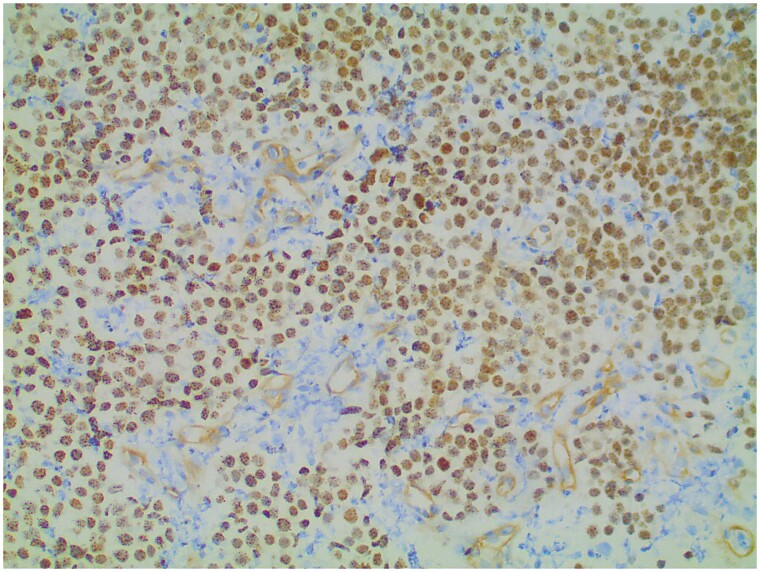
Pathology of NMC. Immunohistochemical stain utilizing clone C52B1. 20×.

The patient is a 25-year-old White male who presented in October 2015 with cough, wheezing, headaches, and 10 days of intermittent hemoptysis. Imaging demonstrated a 5.7 cm mass in the superior segment of the right lower lobe extending into all 3 lobes, associated with significant hilar adenopathy. Bronchoscopy revealed high bronchus intermedius obstruction with involvement of the right upper lobe orifice. Biopsies of the mass showed poorly differentiated high-grade malignant cells with an epithelioid quality (Fig. 1). Immunohistochemistry (IHC) was positive for pan-keratin and cytokeratin 5.2. IHC was negative for CD5, CD30, CD34, CD45, CD117, OCT3/4, SALL4, S100, HMB-45, MART-1, SOX10, p63, high molecular cytokeratin, chromogranin, synaptophysin, NKX2.2, TTF-1, PAX-8, SMA, desmin, TLE-1 and WT-1. Flow cytometry showed no abnormal B- or T-cell populations. Because of the negative results of markers for common tumor types, the patient’s young age, and the finding of a monotonous population of poorly differentiated small round cells testing for NUT protein (Clone C52B1) was carried out. This showed diffuse strong nuclear positivity for NUT protein (CloneC52B1) with a speckled pattern by IHC (Fig. 2). The tumor was negative for *EWSR* rearrangement by fluorescent in situ hybridization (FISH). *EGFR*, *ALK,* and *ROS1* were negative.

**Figure 3.**
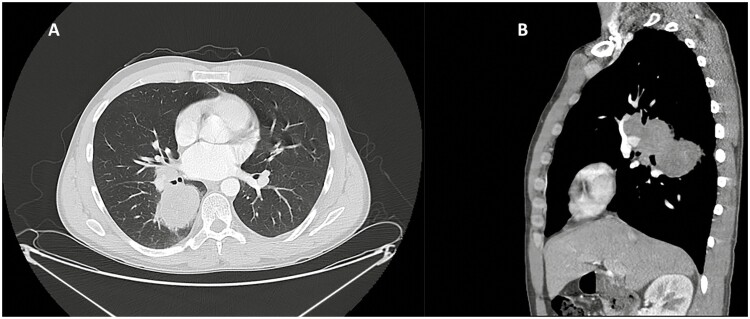
CT scan of chest before surgery. (**A**) Axial view and (**B**) sagittal view.

CTPET showed a 5.2 cm right superior segmental lower lobe mass with a maximum SUV of 9.9 and an adjacent hilar 4.9 cm mass with a maximum SUV of 10.4, but no other foci of concern within the mediastinum and imaged fields (Fig. 3). Brain MRI was normal. Video mediastinoscopy was equivocal for 10R nodal disease, negative in the proximal subcarinal nodes but positive in a distal station 7 node along the bronchus intermedius.

The patient underwent radical intrapericardial right pneumonectomy and regional thoracic lymphadenectomy. Pathology demonstrated a 9.2 cm mass made up of the primary tumor and adjacent involved lymph nodes. Bronchial and vascular margins were positive extraluminally from lymphatic involvement. Multiple hilar nodes were positive. The residual subcarinal nodes were negative but 2 out of 7 4R nodes were positive, the highest node being negative. Genomic analysis was performed on the surgical specimen (TEMPUS) revealing a *BRD4-NUTM1* chromosomal rearrangement. Tumor mutation burden (TMB) was low at 0.8 mutations/megabyte. The tumor was microsatellite stable. RNA sequencing of tumor tissue revealed overexpression of *ERBB2, BRAF, CCND1*, *CCNE1, MAP2K2, mTOR,* and *MYC*. PD-L1 was <1% (Clone 22C3).

Post-operatively, the patient was treated with 6600 cGy with the last 3 fractions delivered to a smaller volume felt to be at the highest risk. Treatment was delivered using a 3-D conformal technique to minimize the dose to the remaining lung. Standard 200 cGy fractionation was used. Treatment was completed in January 2016. The use of systemic chemotherapy and targeted therapy was discussed with the patient. However, the patient elected to forego any therapy, given the lack of definitive data on improvement in survival The patient has undergone surveillance imaging since and he remains free of disease 7.5 years from diagnosis as of March 2023.

## Molecular Tumor Board

### Overview of Nut Midline Carcinoma

NUT midline carcinoma (NMC) is an uncommon malignancy that occurs predominantly in the lung and head and neck regions. This tumor is rare enough that a global registry has been created to collect data and assist patients, their families, and physicians in the care of these patients (www.nmcregistry.org). It presents as a poorly differentiated tumor that requires demonstration of a fusion rearrangement between the *NUTM1* gene and a variety of partners for diagnosis.^1^ The tumor can occur at any age, although it is seen most frequently in adolescents and young adults, with a median age of 24.^2^ Much effort has been invested in determining effective treatment for this disease. However, the outlook for these patients remains extremely poor with a median survival reportedly of 6-7 months_._^2,3^ In a review of 124 cases with a median follow-up of 2.9 years only 6 patients were alive at 5 years and only one was alive at 9 years.^2^

### Pathology and Molecular Characterization

**Figure 4.**
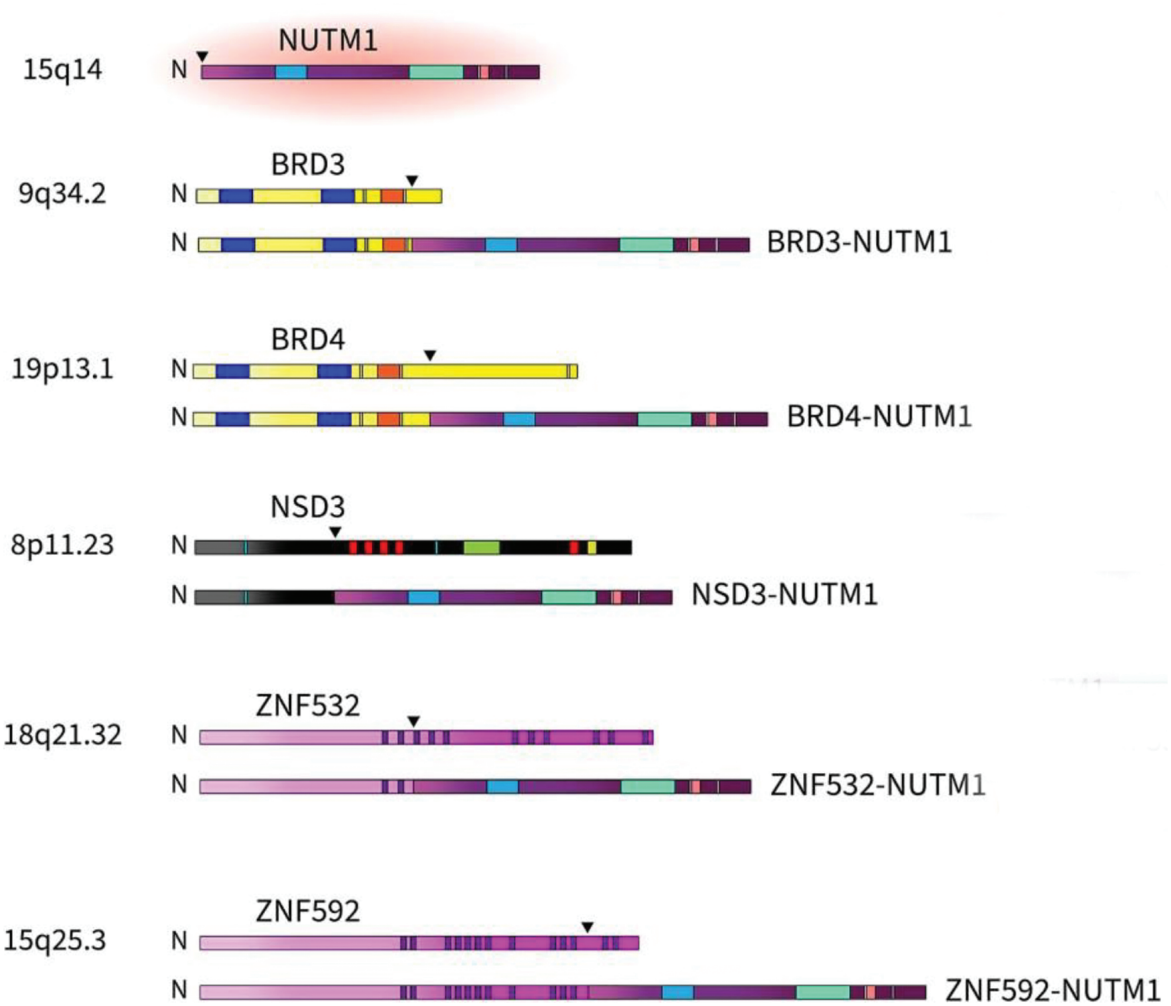
NUTM1 fusions. Schematic of NUTM1 fusions with BRD3, BRD4, NSD3, ZNF532, and ZNF592 and respective wild-type proteins (arrowheads denote fusion breakpoints). The most common of these fusions is BRD4-NUTM1 comprising approximately 70%-80% of all cases. N, amino- or N-terminal, from Moreno et al.^4^

NMC is defined by fusion rearrangements of the *NUTM1* gene, which resides on chromosome 15q14. In approximately 3-quarters of the cases, *NUTM1* is fused with *BRD4*. Other partners include *BRD3, NSD3*, and the zinc finger proteins *ZNF532, ZNF592, ZNF618*, and *ZMYND8* (Fig. 4)^4^. These are poorly differentiated carcinomas that may be mistaken for thymic carcinoma, squamous cell carcinoma, lung cancer, Ewing’s sarcoma, or acute leukemia.^5^ Unlike squamous cell carcinoma, where pleomorphism is seen in the tumor cells, most NMCs demonstrate sheets of monomorphic small to medium undifferentiated round cells with scant-to-moderate amounts of pink-to-clear cytoplasm demonstrating frequent mitoses and single cell or geographic necrosis.^6^ The presence of so-called abrupt foci of keratinization is considered a hallmark of NMC.^7^ ­siRNA-mediated attenuation of *BRD4-NUTM1* expression in vitro has shown that these cells can be induced to differentiate into squamous cells.^5^ The diagnosis of NMC in these poorly differentiated tumors requires demonstration of the appropriate fusion rearrangement. This can be accomplished by immunohistochemistry using a monoclonal antibody to NUT^8^ with confirmation of the fusion *NUTM1*-variant by FISH.^9^ The gene fusion can also be detected with RNA sequencing.^6^

The *BRD4-NUT* fusion which combines the BET protein BRD4 with the NUT protein is the seminal event that results in activation of the histone acetyltransferase p300^1^. This, in turn, leads to hyperacetylation of chromatin, activating transcription of multiple targets, including the ­stem-cell-related transcription factors MYC, SOX2, and TP63. Cellular differentiation is blocked and uncontrolled tumor growth ensues. It is unclear what downstream pathways are up- or downregulated by this enhanced transcription. Advanced sequencing studies and DNA methylation strategies are now being applied in an effort to sort out the epigenetic changes that occur in these tumors.^7,10^

In the study of 124 patients described by Chau et al, none of the patients’ tumors showed evidence of other oncogenic mutations, copy number variation, or genomic rearrangements, although only 10 had molecular genomic profiling, so this must be considered a preliminary finding.^2^ Three groups were identified with prognostically different outcomes: non-thoracic primary and *BRD3*- or *NSD3-NUT* (group A), non-thoracic primary and *BRD4-NUT* (group B), and thoracic primary (group C). Group A had a mOS of 36 months (*n* = 12), group B had mOS of 10 months (*n* = 45), and group C had mOS of 4.4 months (*n* = 67). There were 3/12 5-year survivors in group A, 2/45 in group B, and 0/67 in group C.

In an earlier review of 48 cases of NMC, Chau found that initial surgery ± post-op chemoradiation or radiation alone as well as complete resection with negative margins were significant predictors of overall survival.^11^ The only patients in this cohort who survived 35+ months all initially had surgery. *NUT* translocation type or treatment approaches (including type of chemotherapy regimen) were not associated with survival outcomes. In the more recent larger cohort reported by this group, there were no clear outcome differences by any treatment modality.^2^ There is one report of a 33+ month complete remission with chemotherapy and radiation in a 15-year-old female patient who did not receive surgery for a sinonasal B*RD3-NUT* primary.^12^

### Systemic Therapy

Currently, there are no clearly effective systemic treatments for NMC despite the use of a variety of chemotherapies, BET inhibitors, and histone deacetylase (HDAC) inhibitors. Giridhar^13^ carried out a retrospective review of 119 cases from 64 publications, including the earlier Chau^11^ cohort of 48 patients cited above. Ninety-three of these patients either did not have surgery or there was no information available regarding surgery. Ninety-five patients received chemotherapy with a wide variety of agents. They found that if chemotherapy was included in the initial treatment it improved survival but only in mediastinal tumors. The authors cautioned that the numbers were small and not conclusive.

In vitro and in vivo drug screens have demonstrated the activity of anthracyclines, topoisomerase inhibitors, and microtubule poisons in NMC.^14^ Platinum-based chemotherapy regimens have been tried clinically with modest success.^15,16^ Taxanes, anthracyclines, and a variety of other agents have also been used but there has been no clearly effective regimen. Four groups have reported some success with Ewing’s Sarcoma style regimens, utilizing cyclophosphamide, vincristine, and doxorubicin alternating with ifosfamide and etoposide,^17-20^ while the patient noted above received similar treatment, although without etoposide.^12^ A brief complete response has been reported with gemcitabine.^21^

BET inhibitors have also been studied. These inhibit the binding of BET protein bromodomains to acetylated histones This has been shown to decrease the expression of *MYC*, *SOX2,* and *TP63*. Unfortunately, efficacy has been limited with modest response rates and response durations of only 2-3 months.^1,22^ Multiple mechanisms of resistance to BET inhibitors have been described.^23^ Currently, the BET inhibitor ZEN003694 is being studied in combination with etoposide and cisplatin (NCT05019716). Another trial is utilizing the BRD and BET inhibitors BMS-986158 and BMS-986378 in pediatric cancer patients (NCT03936465). In vitro and in vivo data have suggested that CDK 4/6 inhibitors are synergistic with BET inhibitors in inhibiting NUT carcinoma growth.^24^ NCT05272640 hopes to evaluate the efficacy of ZEN003694 and abemaciclib. HDAC inhibitors have been shown to induce differentiation and growth arrest of NMC cells.^5,25^ Responses have been observed clinically with vorinostat, although the responses have been short-lived and drug tolerance has been an issue.^5,26^

Laboratory data utilizing inhibitors to CDK9 and p300 show some promise.^1,14^ CDK9 inhibition has been shown to be lethal to NMC in vitro along with changes in transcription levels as well as a decrease in *MYC* protein expression.^27^ Wang has demonstrated that the interaction of BRD4-NUT with p300 in NMC cells in vitro results in CDK9-dependent increased phosphorylation of wild-type BRD4 compared to non-NMC cells, leading to increased transcription.^28^ The blockage of phosphorylation by CDK9 inhibition correlated with the repression of downstream oncogenes and abrogation of cellular transformation in the NMC cells. In another study, treatment of NMC cell lines in vitro with NEO02734, a dual inhibitor p300 and BET has shown promise^29^ and is currently recruiting patients in a clinical trial for patients with NMC and prostate cancer (NCT05488548). A number of drugs targeting both CDK9 and *c-MYC* are currently in early-phase clinical trials for a variety of malignancies (www.clinicaltrials.gov).

NMC generally presents with low TMB and negative PD-L1 scores. However, there have been some case reports of high TMB and also with positive PD-L1 scores in NMC. Recent reports have documented responses to immune checkpoint inhibitors (ICI) in patients with both positive and negative PD-L1.^30-33^ There is one report of a 19.5-month response to pembrolizumab^30^ and one of 29 months with nivolumab (with a PD-L1 of 10%).^32^ Riess reported 2 patients who were TMB low and PD-L1 negative who responded to ICIs.^34^ They hypothesized that neoantigens from the gene fusion stimulated host cell cytotoxic T-cell responses, rendering the tumors sensitive to immune checkpoint inhibitors.^35^

Our patient’s tumor showed only the single *BRD4-NUT* fusion mutation, negative PD-L1, and a low TMB, typical of NMC.^30,31^ However, RNA sequencing demonstrated overexpression of multiple potential therapeutic targets. Given the altered transcription that occurs in this disease, this is not surprising.^36^ It will be extremely interesting to see similar studies of DNA, RNA, and proteomics in additional patients.^7,37-43^ It is possible that currently available targeted drugs may be useful in this disease. The use of one targeted drug, anlotinib, has been reported in 2 patients with NMC. This is a receptor tyrosine kinase inhibitor that targets VEGFR1, VEGFR2/KDR, VEGFR3, c-Kit, PDGFR-alpha, and the fibroblast growth factor receptors FGFR1-3.^44^ Chai reported on ongoing 8-month CR to radiotherapy and anlotinib for an orbital NMC.^45^ Jiang treated a patient with pulmonary NMC with radiotherapy plus anlotinib with a short-lived partial response.^46^ We are unaware of reports of this drug being used in the absence of concomitant radiation. Zhou recently reported finding an *NTRK* fusion variant in a patient with a pulmonary NMC. However, larotrectinib was not yet available for use in this case.^47^

## Conclusion

NMC is a very uncommon disease that usually presents in younger patients in either the lung or head and neck regions. It requires specific diagnostic tools with either IHC, FISH, or genomics to confirm the diagnosis and thus a high index of suspicion. Thus far, while a variety of systemic treatments have been tried, their efficacy has been quite limited. Effective surgery and radiation remain the cornerstones of treatment, as demonstrated by the remarkable survival of the patient reported here. Additional evaluation of ICI therapies is needed. Further studies of the epigenetics of this tumor may uncover druggable targets that could be useful.

## Data Availability

The data underlying this article will be shared on reasonable request to the corresponding author.
